# Short-term heat waves have long-term consequences for parents and offspring in stickleback

**DOI:** 10.1093/beheco/arae036

**Published:** 2024-04-27

**Authors:** Rachel Barrett, Laura R Stein

**Affiliations:** School of Biological Sciences, 730 Van Vleet Oval, Rm 314, University of Oklahoma, Norman, OK, United States; School of Biological Sciences, 730 Van Vleet Oval, Rm 314, University of Oklahoma, Norman, OK, United States

**Keywords:** heat wave, parental care, parental effects, plasticity, stickleback

## Abstract

Extreme temperature events, such as heat waves, can have lasting effects on the behavior, physiology, and reproductive success of organisms. Here, we examine the impact of short-term exposure to a simulated heat wave on condition, parental care, and reproductive success in a population of threespine stickleback (*Gasterosteus aculeatus*), a small fish with exclusive paternal care, currently experiencing regular heat waves. Males were either exposed to a simulated heat wave (23 °C) for 5 d or held at an ideal temperature (18 °C). Following this 5-d treatment, all males were transferred to 18 °C, where they completed a full parenting cycle. Offspring were raised at 18 °C. We found that while mass and body condition were unaffected in males exposed to a heat wave, cortisol responses were dampened across the nesting cycle compared to control males. In addition, heat wave males had longer latency for eggs to hatch, lower hatching success, and showed lower levels of parental care behavior compared to control males. Offspring of heat wave males had lower body condition, affecting swimming performance. Altogether, our results highlight the long-term impact that even short-term events can have on reproductive success, parental behavior, and subsequent generations, providing insight into population responses to rapid environmental change.

## Teaser

Extreme weather events, such as heat waves, are increasing in frequency and severity. While the consequences of increased ambient temperatures are well-documented, less is known about long-term impacts after conditions have returned to normal. In threespine stickleback, a cold-water fish with paternal care, fathers have reduced reproductive success and are unable to parent at full capacity well after the heat wave has concluded. This affects the offspring’s condition and behavior, highlighting the long-term impact of short-term events.

## Introduction

Behavior shapes species’ persistence and resilience because it is often an individual’s first response to environmental change (Wong and Candolin 2015), with the potential to buffer against or accelerate responses to climate change or habitat disturbance; for example, adjustments to parental care may protect offspring from heat or weather events (Grew et al. 2019), while evolutionary traps such as laying eggs on asphalt instead of water reduce reproductive success (Sih 2013; Wong and Candolin 2015). Behavioral plasticity, or the ability of organisms to flexibly adjust their behavior in response to the environment, has been proposed as a major mechanism for predicting species survival in the face of climate change (Purvis et al. 2000; Sih 2013; Maspons et al. 2019).

In species with parental care, parenting behaviors act as a mediator between offspring and their environment. To maximize fitness and offspring success, parents should adjust their care behavior in response to environmental conditions (Klug and Bonsall 2014; Clutton-Brock 2019). Additionally, while parental behavior is predicted to buffer offspring against stressors and increase reproductive success via direct impacts on offspring survival (Klug and Bonsall 2014; Kölliker et al. 2014; Clutton-Brock 2019), changes in care in response to environmental stressors can also have important subsequent effects on offspring development and fitness (Candolin et al. 2008; Stein and Bell 2014; Fox et al. 2018; Grew et al. 2019; Tariel et al. 2020; MacLeod et al. 2022). Parental care can be a key mechanism by which parents influence offspring development and behavior via parental effects—if parents can both compensate for stressful conditions and produce adaptive offspring phenotypes, this may increase offspring success and have important implications for population resilience (Lindström, 1999; Badyaev and Uller 2009; Donelson et al. 2018; Bell and Hellmann 2019; Donelan et al. 2020). Understanding how changes in parental care in response to the environment impact offspring development is critical to understanding both the evolution of parental care behavior and how organisms might respond to environmental change.

To date, the vast majority of theoretical and empirical work on plastic responses to climate change has focused on within-generational plasticity (Wong and Candolin 2015). However, across-generational plasticity (parental effects, or the parental ability to convey information or non-genetically altered development across generations) may play a key role in how organisms respond to rapidly changing environments (Donelson et al. 2018; Donelan et al. 2020). Parental effects might be especially relevant in understanding the biological impacts of climate change because some changes, such as increases in heat, will persist across generations for nearly all species, allowing parents to provide offspring with information regarding the environment they will likely encounter. On the other hand, parental effects might be less potent under climate change due to increased environmental variability, limiting predictability. If parental effects are adaptive, they can buffer populations against the immediate effects of climate change and provide time for genetic adaptation to catch up in the longer term (Chevin et al. 2010; Kopp and Matuszewski 2014; Leimar and McNamara 2015; Donelson et al. 2018; Donelan et al. 2020). Alternatively, parental effects may be maladaptive if parents cannot either meet energy requirements for parenting or sufficiently change their behaviors in response to changing offspring needs under rapidly changing conditions (Schuler and Orrock 2012; Kuijper and Johnstone 2016; Donelson et al. 2018; Donelan et al. 2020; Lind et al. 2020).

Threespine stickleback (*Gasterosteus aculeatus*) are a small teleost fish wherein males are the sole provider of care, and parenting is necessary for offspring survival (Wootton 1984). Males build a nest and vigorously court females. Once a female has spawned, she leaves and the male cares for eggs by defending the nest against predators, fanning water through the nest, and tending the nest (Wootton 1984; Smith and Wootton 1999). Embryonic development changes in response to pH, dissolved oxygen (DO), and temperature (Glippa et al. 2017). Fathers increase fanning behavior in response to increases in ambient temperature (Hopkins et al. 2011; Isotalo et al. 2022) and lowered DO/eutrophication (Candolin et al. 2008; Fox et al. 2018), although this does not always lead to an increase in reproductive success (Fox et al. 2018). Therefore, fathers may adjust their behavior in response to both their own energetic needs and alterations to offspring development under adverse conditions.

Here, we report how short-term exposure to a simulated heat wave can impact future reproductive success and offspring development and behavior, even after the extreme event has ended. We tested the hypothesis that a short-term exposure to high water temperature would alter paternal care in threespine stickleback, even when returned to ideal conditions, and this, in turn, would affect offspring. Previous studies have demonstrated that when held under long-term elevated temperatures while parenting, stickleback males had lower reproductive success and higher rates of fanning (Hopkins et al. 2011; Isotalo et al. 2022). Here, we highlight the effects of a short-term exposure to a simulated heat wave and its impacts on parental care after this stressful event has already concluded. Considering that reproduction in threespine stickleback is already a costly energetic investment for males (Smith and Wootton 1999), we predicted that exposure to a simulated heat wave would reduce body condition after parenting, increase the amount of time it takes for a male to initiate the reproductive behavior cycle, increase cortisol levels as a proxy for physiological stress response (Pankhurst 2011; Jessop et al. 2016; de Bruijn and Romero 2018; Mentesana and Hau 2022), and decrease parenting behaviors, ultimately resulting in negative impacts on offspring development and cascading into effects on offspring behavior.

## Methods

### Fish site, collection, and husbandry

We collected young-of-the-year juvenile stickleback from the Navarro River in Mendocino County, CA (39.098173569308, -123.49980254730339) in August 2021 and shipped to our colony at the author’s home institution. The Navarro River is an undammed river in northern California and has been experiencing intense effects of long-term drought, including frequent heatwaves (Thompson et al. 2022). Standard water temperatures range from 15 to 19 °C in the summer, but heat waves induce temporary spikes of up to 26 °C (U.S. Environmental Protection Agency (USEPA) 2000, pers. measurements). In June 2021, this region of northern California (along with the rest of western North America) experienced an unprecedented, record-breaking heat wave over 5 d (Philip et al. 2022; Thompson et al. 2022). We collected approximately 1-mo-old stickleback at the end of the breeding season, and there had been no recorded heatwave in the prior month; therefore, these individuals would not have yet parented nor been exposed to heat. Stickleback were held at 18 °C water temperature with a 9:15-h winter (November 1 to March 31) light cycle and 15:9-h summer (April 1 to October 31) light cycle on recirculating racks (Aquaneering) that replaces water in each tank multiple times per day. Stickleback require a winter cycle for spermatogenesis and to stimulate breeding in the summer (Borg 1982; Sattler and Boughman 2020). 18 °C was chosen as a baseline temperature as this is ecologically relevant for this population (U.S. Environmental Protection Agency (USEPA), 2000), falls within the natural temperature range for this species of 4 to 20 °C (Allen and Wootton 1982), and represents a growth optimum (Allen and Wootton 1982; Dittmar et al. 2014). Each rack has a chiller that allows for precise manipulation of temperature. Males and females were kept in groups in 75.7 L tanks. All fish were fed a mixture of frozen-thawed bloodworms and mysis shrimp *ad lib* each day, with live *Artemia* shrimp additionally fed every other day. Reproduction in this species requires males to build nests; therefore, the lack of nesting material in these group tanks ensured that males did not parent until the experiment began.

### Temperature treatment

**Fig. 1.**
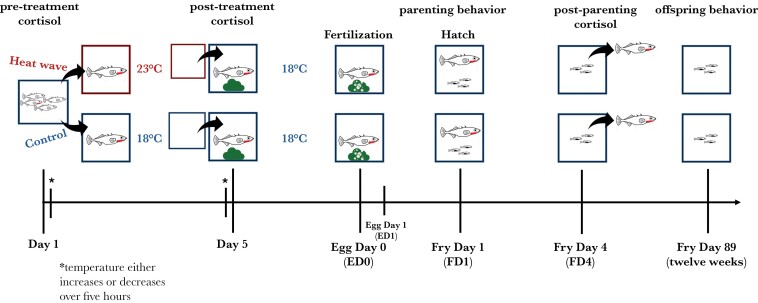
Experimental design. Males were held in the lab at 18°C in mixed sex tanks until the start of the experiment. Males in reproductive coloration (red throat) were randomly selected and assigned to either the heat-wave or control treatment. Fish were placed in individual tanks and heat-wave males were held at 23 °C for 5 d, while control males were held *n* = 22 control, *n* = 27 heat. Significant effects (*P* ≤ 0.05) indicated in bold at 18 °C. After 5 d, all males were transferred to individual tanks with material for nesting at 18 °C. Males were presented with females once nests were completed, and parental behavior was observed daily until 4 d after fry hatched. On the fifth day after hatch, males were removed and offspring reared to 12 wk, at which time they were measured and behaviorally assayed. Stickleback line drawings by M. Bensky.

At the start of the experiment in May 2022, males (identified by nuptial coloration) were weighed, their length was measured, and they were held for “pretreatment” cortisol (see *Cortisol measurements* below). Males were then randomly assigned to one of two treatment groups, heat wave or control, and separated into 43.2 L individual tanks (28 × 50.8 × 30.5 cm) with gravel, a black plastic “plant,” and black shaders on the sides and back of the glass to prevent visual contact with other males (Fig. 1). The heat wave group was placed at 18 °C and temperature gradually increased to 23 °C over the course of 5 h. Males were then held at 23 °C for a 5-d period, while the control group was held at 18 °C during this time (Fig. 1). 23 °C was chosen as the stressful temperature for the heat wave treatment as it is ecologically relevant, with the Navarro River reaching 23 °C during heat waves (U.S. Environmental Protection Agency [USEPA] 2000), continuous exposure reduces stickleback immune response (Dittmar et al. 2014) and reproductive success (Hopkins et al. 2011; Fox et al. 2018), but is below their upper thermal tolerance threshold (Barrett et al. 2011; Dittmar et al. 2014). After 5 d, males in the heat wave were transferred back from 23 °C to 18 °C over the course of 5 h, all fish were removed from their tanks, weighed, measured for length and cortisol as before, and placed in a new, randomized individual tank at 18 °C. Each male was provided with nest-building materials: a plastic container (8 × 8 × 3 cm) filled with fine white playground sand and 4 g of filamentous algae. Our final sample size was *n* = 18 control and *n* = 18 heat males. There was zero mortality in either treatment group during the treatment application.

### Parenting observations

After nest completion (identified by a characteristic “tunnel” opening), males were presented with gravid females (identified by characteristic egg visibility at cloaca) from the group tank (Wootton 1984). Female masses were taken before and after egg laying to approximate egg mass. Male behaviors were measured daily from one day after spawning (ED1) until 4 d after the eggs had hatched (FD4), when fry are independent and males in this population leave their nests. Parental behaviors (time at nest, time fanning) were recorded for 5 min every day, following Bell et al. (2018) and Hellmann et al. (2021). Males were observed between 0800 and 1200 from behind a blind in real time and observers (LRS and RB) were blinded to male treatment. Five days after eggs hatched (FD5), the male was removed, final mass and length were recorded as above, and cortisol measurements were taken. We counted the number of offspring and relocated them to a new 21.8 L tank (14 × 50.8 × 30.5 cm) containing gravel, a plastic “plant,” and a yarn mop. Each clutch of offspring was reared with their sibling group. Due to variations in willingness to build a nest, nest abandonment, and clutch success, our final sample size for parenting behavior was *n* = 10 control and *n* = 11 heat wave males. Due to differences in time to hatch, total time parenting (ED1—FD4) ranged from 9 to 15 d (mean 10.10 ± 0.14 d).

### Cortisol measurements

Cortisol is a hormone often associated with stress (Sadoul and Geffroy 2019). Threespine stickleback, like all fishes, excrete excess cortisol via their gills and this correlates to circulating plasma cortisol (Sadoul and Geffroy 2019), allowing for noninvasive multiple measurements of the same individual. Cortisol was measured at 3 time points: immediately before and immediately following the simulated heat wave (or control), and after parenting concluded (5 d following fry hatch). Individual males were placed in an opaque plastic cup containing 50 mL of fresh water for 30 min in an isolated room. The male was removed, and water was held at −20 °C until analysis. This took place immediately before measuring the weight and length of males, and males were returned to their individual tanks once all measurements were taken.

We extracted cortisol from water samples following (Sadoul and Geffroy 2019). Briefly, samples were thawed at room temperature, passed through 5 mL C18 methanol-activated cartridges (Sep-Pak, Waters Inc.) through a vacuum pump, and rinsed with 10 mL ultra-pure water at 10 mL/minute. The cartridge was then eluted into a glass test tube with 10 mL of methanol and evaporated under nitrogen. We reconstituted the samples with 400 μL of assay buffer, vortexed the sample twice, and transferred the sample to microcentrifuge tubes. Cortisol samples were diluted 25-fold with ELISA buffer concentrate (1×) and ran in triplicate on an ELISA plate following standard protocols (Cayman Chemical). The plate was read at 450 nm, and measurements were assessed via a standard curve. We report cortisol as a release rate (ng/g/min), following Sebire et al. (2007) and Sadoul and Geffroy (2019)). A total of *n* = 6 plates were used; intra-assay CV averaged 4.9 ± 1.08%; inter-assay CV was 9.7%.

### Offspring measurements and scototaxis assay

Fry were fed *Artemia* brine shrimp every other day. After fry reached 12 wk of age, we performed a scototaxis assay to evaluate behavioral differences between father treatments. Scototaxis assays are used to assess exploratory and anxiety behaviors in a wide variety of taxa, where time on the black side reflects anxiety-related behaviors (Maximino et al. 2010). Previous work in stickleback suggests that offspring receiving sub-standard care show greater anxiety behavior (McGhee and Bell 2014). The assay consisted of a 13.2 L bucket covered half in black duct tape (including the sides) and half in white duct tape. Fish were transferred individually to the assay in an opaque cylinder (10 × 10 cm) plugged with a rubber stopper. This “pod” was placed in the center of the arena, with the opening facing the middle between the white and black sides. Following a 5-min acclimation period, the stopper was removed via fishing line, and the assay was recorded from above with a camera (DJI Osmo Action). Individuals were given 3 min to emerge from the pod, and latency to emerge (time from stopper removed to tail fully emerged) was measured. If they did not emerge within 3 min, individuals were given the maximum latency to emerge (181s), gently released into the assay by slowly inverting the pod until the fish was fully in the arena, the pod was placed back into its original position, and we recorded behaviors for five minutes. Following the assay, individuals were measured for length and mass and transferred back into their home tank. We recorded time in the pod, time swimming, and time either on the black or white side of the bucket from recorded video using BORIS software (Friard and Gamba 2016). The coder (XX) was blinded to the offspring’s family and paternal treatment. Our final sample size was *n* = 22 offspring of control fathers (*n* = 10 families) and *n* = 27 offspring of heat wave fathers (*n* = 11 families).

### Statistical analysis

Data were analyzed using R studio 2021.09.0. Data and code are available in Supplementary Information. Residuals were examined for normality prior to all analyses. Unless indicated otherwise, we used linear mixed models with a normal distribution (analyzed with the lmerTest package (Kuznetsova et al. 2017), and significance between treatment levels was determined using emmeans from the emmeans package (Lenth et al. 2018). All residuals were inspected for violations of model assumptions; if violations were found, we transformed our data and reexamined the residuals. For male mass and body condition, we included treatment (control, heat wave), timepoint (pretreatment, post-treatment, and post-parenting), and their interaction as fixed effects, and individual ID as a random effect to account for multiple measurements of the same individual. Mass was natural-log transformed prior to analysis. We calculated body condition using Fulton’s body condition factor *K*, a suitable indicator of body fat content in small fish (Kotrschal et al. 2011). Fulton index *K* is calculated as *K = M/SL*^3^ *100,000 as in (Kotrschal et al. 2015), where *M* is the fish’s body mass (g), and *SL* is its standard length (mm) (Bolger and Connolly 1989).

Linear mixed models were used to assess cortisol similar to mass and body condition, including “ELISA Plate” in the random effects to account for interassay variation. Cortisol was divided by mass at the time of measurement and by time in the cup (30 min) to obtain ng/g/min (Sebire et al. 2007; Sadoul and Geffroy 2019). Three samples were outside the standard curve (1 control post-parenting, 1 heat post-treatment, and 1 heat post-parenting) and were excluded from analysis.

For latency to build a nest, latency to hatch, and hatching success (residuals from the number of eggs hatched in relation to egg mass), we ran linear models with treatment (control, heat wave) as a fixed effect and body condition as a covariate. Here, we used linear models instead of linear mixed models, as each male only had one measurement, and we did not need to account for multiple measurements of the same individual via random effects. To assess hatching success, we calculated residuals from the number of fry hatched regressed on initial egg mass. As stickleback fertilize eggs inside the nest and may abandon if the nest is disturbed, we could not count the initial egg number. Latency to hatch was natural-log transformed prior to analysis. One male (treatment: heat wave) took 38 d to build a nest; this data point was excluded from our latency to build a nest model.

To assess whether paternal behavior was influenced by treatment across the nesting cycle, we ran linear mixed models separately for the time at the nest and percent time fanning while at nest with treatment, nesting stage (ranging from one day post-fertilization to 4 d post-hatching), and their interaction as fixed effects, post-treatment body condition and egg mass as covariates, and observer and individual ID as random effects.

Finally, offspring length, mass, and body condition were analyzed via linear mixed models with paternal treatment (control, heat wave) as a fixed effect, rearing density as a covariate, and father ID as a random effect to account for multiple offspring measured from the same family. Offspring behavior (time in pod, time in white, time swimming) were each analyzed with linear mixed models with paternal treatment as a fixed effect, rearing density as a covariate, and father ID as a random effect to account for multiple offspring measured from the same family. Body condition was square-root transformed prior to analysis. As body condition was influenced by paternal treatment, any differences in behavior could be cofounded. Therefore, we assessed whether body condition influenced each offspring behavior separately. We found that body condition affected time swimming; therefore, we analyzed residuals from time swimming in relation to body condition.

We report marginal *R*^2^ values for our mixed models as a measure of fixed effect sizes. Marginal *R*^2^ values describe the variance explained by fixed factors, while conditional *R*^2^ describes the variance explained by both fixed and random factors. We report η^2^ (eta^2^) values as a measure of fixed effects for our linear models. η^2^ values are calculated based on the sum of squares and provide an estimate of the proportion of variation associated with a given factor. All data and code are available on Dryad (Barrett and Stein 2024).

### Ethical note

All methods were approved under University of Oklahoma IACUC R20-008, and fish were collected under California Department of Fish and Wildlife specific use permit S-192960002-21067-001 to LRS. We used power analyses to identify the minimum number of animals while maintaining statistical integrity, and our sample sizes are comparable to similar studies on parenting behavior in stickleback (Stein and Bell 2012; Bell et al. 2018; Hellmann et al. 2021). Housing conditions in the laboratory adhered to the most recent ASAB/ABS Guidelines at the time of data collection (“Guidelines for the treatment of animals in behavioural research and teaching” 2021) for the treatment of animals in behavioral research and teaching. Fish were kept in natural social groups and tanks were enriched with gravel, hides (plastic plants and yarn mops), and materials for nest building for males. Our maximum heat in the heat wave treatment does not exceed threespine stickleback thermal tolerance of 26 to 30 °C (Allen and Wootton 1982; Dittmar et al. 2014; Schade et al. 2014). For behavioral assays and measurements, individuals were gently but quickly netted and transferred in opaque containers, and assays were recorded either from behind a blind in their home tanks (parenting) or in a separate room without a live observer (scototaxis) to minimize stress. Light cycles and water chemistry mimicked natural conditions as closely as possible, and daily health and water quality checks were conducted.

## Results

### Heat wave effects on male mass, body condition, and cortisol

**Fig. 2.**
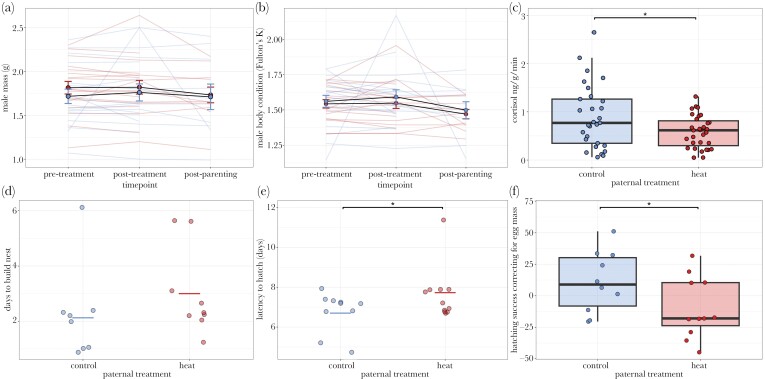
Effects of a simulated heat wave on male condition, cortisol, and life history traits. For all graphs, points are individuals. A) Male mass was unaffected by a simulated heat wave and parenting. Lines represent individual males; filled circles and error bars represent means and standard errors. B) Male body condition (Fulton’s K, g/mm^3^ * 100,000) was unaffected by a simulated heat wave and parenting. Lines represent individual males; filled circles and error bars represent means and standard errors. C) Males exposed to a heat wave had lower cortisol than control males. D) There was no difference between treatments in number of days it took males to build a nest. Horizontal bars indicate means. E) Embryos of heat wave males took longer to hatch than embryos of control males. Horizontal bars indicate means. F) Hatching success (residuals of number of fry hatched regressed on initial egg mass) was greater in control than heat wave males.

Male mass was unaffected by treatment (*F*_1,34.79_ = 0.016, *P* = 0.90), timepoint (*F*_2,56.36_ = 0.46, *P* = 0.64), or their interaction (*F*_2,54.73_ = 0.35, *P* = 0.71) (Fig. 2A; Table 1). Similarly, body condition did not change across treatments (*F*_1,35.54_ = 0.28, *P* = 0.60), timepoint (*F*_2,56.41_ = 2.57, *P* = 0.08), or their interaction (*F*_2,56.41_ = 0.14, *P* = 0.87) (Fig. 2B; Table 1).

**Table 1.** Linear mixed model results for paternal mass, body condition, and cortisol.

Factor	Mass (g)			Body condition (Fulton’s *K*)			Cortisol (ng/g/min)		
	*F*	*df*	*P*	*F*	*df*	*P*	*F*	*df*	*P*
Treatment	0.016	1,34.79	0.90	0.27	1,35.54	0.60	**5.85**	**1,17.62**	**0.03**
Stage	0.46	2,56.36	0.64	2.57	2,56.28	0.09	0.25	2,33.87	0.78
Length	**175.54**	**1,57.87**	**<0.0001**				0.87	1,24.71	0.36
Treatment × Stage	0.35	2,54.73	0.71	0.14	2,56.28	0.87	0.05	2,33.82	0.95
**Random effect**	LRT		*P*	LRT		*P*	LRT		*P*
Individual ID	**27.62**		**<0.0001**	**11.08**		**0.0009**	0.23		0.63
ELISA Plate							3.19		0.07
Marginal *R*^2^	0.75			0.04			0.09		
Conditional *R*^2^	0.90			0.43			0.33		
Log-likelihood	117.14 (*df* = 9)			29.35 (*df* = 8)			−47.22 (*df* = 10)		

For mass and body condition, sample sizes were as follows: pretreatment: *n* = 18/treatment, post-treatment: *n* = 18/treatment, post-parenting: *n* = 10/control, *n* = 11/heat. For cortisol, sample sizes were as follows: pretreatment: *n* = 10/control, *n* = 11/heat; post-treatment: *n* = 10/control, *n* = 10/heat; post-parenting: *n* = 9/control, *n* = 10/heat. Significant effects (*P* ≤ 0.05) indicated in bold.

The cortisol release rate was affected by treatment (*F*_1,17.61_ = 5.86, *P* = 0.03) (Fig. 2C; Table 1), where males exposed to a heat wave had lower cortisol release rate overall (mean ± SE; 0.59 ± 0.06 ng/g/min) than control males (0.90 ± 0.12 ng/g/min). While there was no significant interaction between treatment and timepoint, we wished to examine patterns of cortisol across all sampling timepoints. Cortisol was highest in control males 5 d after being held in individual tanks (post-treatment) (1.04 ± 0.18 ng/g/min).

### Heat wave effects on male life-history traits and parenting behavior

Neither a simulated heat wave (*F*_1,14_ = 1.07, *P* = 0.32) nor body condition (*F*_1,14_ = 0.85, *P* = 0.37) affected latency to complete nest building among males (Fig. 2D; Table 2). However, males exposed to a heat wave differed from control males (*F*_1,18_ = 5.00, *P* = 0.04), wherein embryos of heat wave males took longer to hatch (7.73 ± 0.36 d) than embryos of control males (6.70 ± 0.30 d) (Fig. 2E; Table 2). We found a difference between treatments in hatching success (*F*_1,18_ = 4.30, *P* = 0.05), such that males exposed to a heat wave had fewer eggs hatch than expected by egg mass (−9.94 ± 7.33) than control males (10.94 ± 7.65) (Fig. 2F; Table 2).

**Table 2.** Linear model results for days to nest, latency to hatch, and hatching success (corrected for egg mass).

Factor	Days to nest				Latency to hatch				Hatching success (corrected for egg mass)			
	*F*	*df*	*P*	η^2^	*F*	*df*	*P*	η^2^	*F*	*df*	*P*	η^2^
Treatment	1.07	1,14	0.32	0.07	**5.00**	**1,14**	**0.04**	**0.21**	**4.29**	**1,18**	**0.05**	**0.17**
Body condition	0.85	1,14	0.37	0.05	0.62	1,14	0.44	0.03	3.04	1,18	0.10	0.12

*n* = 10 control, *n* = 11 heat. Significant effects (*p* ≤ 0.05) indicated in bold.

**Fig. 3.**
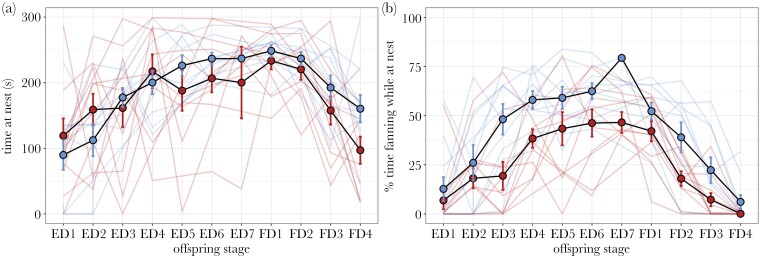
Effects of a simulated heat wave on paternal behavior. Lines represent individual males; filled circles and error bars represent means and standard errors. Blue (light) indicates control males, red (dark) indicates males exposed to a simulated heat wave. ED represents days post-fertilization, FD represents days post-hatch. A) Males adjust time at nest based on embryo age (stage), but not treatment. B) Males adjust percent time fanning at nest based on both embryo age (stage) and treatment, where males exposed to a simulated heat wave fan less than control males.

Exposure to a heat wave did not affect time spent at the nest (*F*_1,21.21_ = 0.49, *P* = 0.49), but males adjusted time at the nest across the nesting cycle (*F*_10,167.38_ = 10.85, *P* < 0.0001) (Fig. 3A; Table 3). Similarly, males adjusted percent time fanning at the nest across the nesting cycle (*F*_10,169.32_ = 23.02, *P* < 0.0001). We observed a steady increase and a peak in both parenting behaviors at egg days 5–7, just prior to offspring hatch, followed by a decrease as fry aged, similar to previous observations for this species (Stein and Bell 2012; Bell et al. 2018; Hellmann et al. 2021). In addition, exposure to a heat wave changed percent time fanning (*F*_1,22.59_ = 14.51, *P* = 0.0009), such that heat wave males overall fanned less while at the nest (24.22 ± 2.21 %-time fanning) than control males (38.34 ± 2.72 %-time fanning) (Table 3). This difference appeared 3 d post-fertilization (ED3) and remained across the nesting cycle (Fig. 3B).

**Table 3.** Linear mixed model results for time at nest (s) and % time fanning while at nest.

Factor	Time at nest (s)			% time fanning while at nest		
	*F*	*df*	*P*	*F*	*df*	*P*
Treatment	0.49	1,21.21	0.4	**14.51**	**1,22.59**	**0.0009**
Stage	**10.85**	**10,167.38**	**<0.0001**	**23.02**	**10,169.32**	**<0.0001**
Egg mass	4.11	1,16.65	0.06	0.62	1,18.10	0.44
Body condition	2.98	1,16.10	0.10	1.90	1,16.82	0.19
Treatment × Stage	1.35	10,168.54	0.21	0.96	10,169.51	0.48
**Random effect**	LRT		*P*	LRT		*P*
Individual ID	**5.44**		**0.02**	**5.06**		**0.02**
Observer	0.04		0.85	0		1
Marginal *R*^2^	0.37			0.54		
Conditional *R*^2^	0.44			0.60		
Log-likelihood	−1058.42 (*df* = 27)			−817.68 (*df* = 27)		

Sample sizes were *n* = 10 control, *n* = 11 heat. Significant effects (*P* ≤ 0.05) indicated in bold.

We found no relationship between time nesting post-treatment and average percent time fanning (that is, males initiating reproduction later did not have a greater recovery in fanning behavior) (Pearson correlation: *r*^2^ = 0.01, 95% CI [−0.42, 0.44]). We additionally found no correlation between average percent time fanning and either latency for eggs to hatch (Pearson correlation: *r*^2^ = −0.16, 95% CI [−0.55, 0.29]) or hatching success (Pearson correlation: *r*^2^ = 0.25, 95% CI [−0.18, 0.60]).

### Effects of paternal experience with heat waves on offspring morphology and behavior

**Fig. 4.**
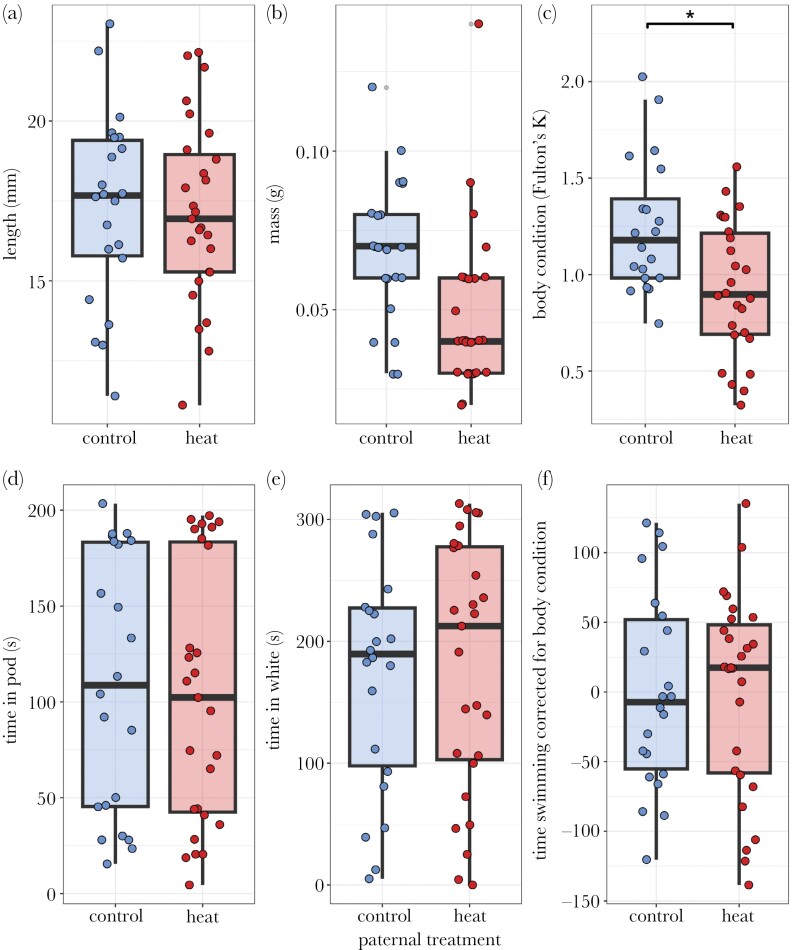
Juvenile offspring condition and behavior in a scototaxis assay in relation to paternal treatment. For all graphs, points are individuals. A) Offspring did not differ in size based on paternal treatment. B) Offspring did not differ in mass based on paternal treatment. C) Offspring differed in body condition (Fulton’s *K*, g/mm^3^ * 100,000) based on paternal treatment, such that offspring of fathers exposed to a heat wave were in worse condition than offspring of control fathers. D) Offspring did not differ in time spent in the pod before emerging into a novel environment based on paternal treatment. E) Offspring did not differ in time spent in white in a scototaxis assay based on paternal treatment. F) When correcting for body condition, offspring did not differ in time swimming in a scototaxis assay based on paternal treatment.

Offspring of heat wave and control males did not differ in length (*F*_1,10.59_ = 1.12, *P* = 0.31) or mass (*F*_1,8.51_ = 0.003, *P* = 0.96) (Fig. 4A,B; Table 4). We found an effect of rearing density on mass (*F*_1,7.16_ = 6.19, *P* = 0.04), where offspring of larger clutches had less mass. Paternal exposure to a heat wave did influence offspring body condition (*F*_1,46_ = 6.31, *P* = 0.02), with offspring of heat-exposed fathers in worse condition (0.99 ± 0.09 g/mm^3^ × 100,000) than offspring of control fathers (1.40 ± 0.13 g/mm^3^ × 100,000), regardless of rearing density (*F*_1,46_ = 0.16, *P* = 0.69) (Fig. 4C; Table 4).

**Table 4.** Linear mixed models for offspring length, mass, and body condition.

Factor	Length (mm)			Mass (g)			Body condition (Fulton’s *K*)		
	*F*	*df*	*P*	*F*	*df*	*P*	*F*	*df*	*P*
Paternal Treatment	1.12	1,10.59	0.31	0.003	1,8.51	0.96	**5.80**	**1,7.25**	**0.05**
Density	3.75	1,7.37	0.09	**6.19**	**1,7.16**	**0.04**	0.04	1,3.81	0.86
**Random Effect**	LRT		*P*	LRT		*P*	LRT		*P*
Paternal ID	0.71		0.40	**3.79**		**0.05**	0.09		0.76
Marginal *R*^2^	0.10			0.30			0.17		
Conditional *R*^2^	0.20			0.55			0.21		
Log-likelihood	−224.79 (*df* = 5)			103.71 (*df* = 5)			275.11 (*df* = 5)		

*n* = 22 control, *n* = 27 heat. Significant effects (*P* ≤ 0.05) indicated in bold.

We found no effect of paternal treatment on time in the pod (*F*_1,9.32_ = 1.34, *P* = 0.28) (Fig. 4D), time in white (*F*_1,11.89_ = 1.14, *P* = 0.31) (Fig. 4E), or time swimming (*F*_1,9.38_ = 0.41, *P* = 0.54) (Fig. 4F) of offspring in the scototaxis assay (Table 5). Rearing density influenced time in the pod (*F*_1,4.34_ = 7.37, *P* = 0.049), where offspring reared at higher densities spent less time in the pod (Supplementary Fig. S1). Offspring body condition influenced time swimming (*F*_1,41.70_ = 5.88, *P* = 0.02), where offspring in better condition swam less (Supplementary Fig. S2). As offspring body condition was dependent on paternal treatment (Fig. 4C), we ran an additional model using residuals from time swimming regressed on body condition, with treatment as a fixed effect, rearing density as a covariate, and paternal ID as a random effect. This did not change our results; there remained no overall effect of paternal treatment (*F*_1,8.39_ = 0.40, *P* = 0.55; Fig. 4F).

**Table 5.** Linear mixed models for offspring behavior in a scototaxis assay.

Factor	Time in pod (s)			Time in white (s)			Time swimming corrected for body condition		
	*F*	*df*	*P*	*F*	*df*	*P*	*F*	*df*	*P*
Paternal Treatment	1.81	1,7.79	0.22	2.33	1,10.70	0.16	0.40	1,8.39	0.55
Density	**7.67**	**1,4.28**	**0.05**	4.41	1,7.61	0.07	0.47	1,6.61	0.52
**Random effect**	LRT		*P*	LRT		*P*	LRT		*P*
Paternal ID	0.004		0.95	0.94		0.33	1.90		0.17
Marginal *R*^2^	0.14			0.12			0.02		
Conditional *R*^*2*^	0.15			0.24			0.26		
Log-likelihood	-262.11 (df = 5)			-279.67 (df = 5)			-274.78		

*n* = 22 control, *n* = 27 heat. Significant effects (*P* ≤ 0.05) indicated in bold.

## Discussion

Anthropogenic climate change has been attributed to the increasing frequencies and intensities of extreme weather events, including droughts, wildfires, hurricanes, flooding, and heat waves (Mills 2009). Heat waves, in particular, have intensified in many regions of the world (Cueto et al. 2010; Peterson et al. 2013; Lhotka et al. 2018). Aquatic systems are especially vulnerable to heat waves, as higher water temperatures surpass the temperature threshold of many aquatic organisms, including fishes (Häder and Barnes 2019). Some aquatic systems, such as lakes or intermittent streams and rivers (such as our study population), are locked within one region due to physical barriers, meaning that fish in these habitats are incapable of migrating away from extreme conditions and must navigate living under poor conditions of their existing environment (Davidsen et al. 2020). Here, we investigated the impacts of a simulated heat wave on parental condition, cortisol, behavior, and reproductive success in a population of threespine stickleback currently experiencing drought and heat waves. We additionally measured offspring condition and behavior. We found that a simulated heat wave suppressed paternal care behaviors, such that males exposed to a heat wave were not able to fan their nests at the same rate as control individuals. In addition, heat wave males had an increased latency for their eggs to hatch, decreased hatching success, and suppressed cortisol response across the nesting cycle. Offspring of heat-exposed males were in worse body condition at 3 mo, which corresponded to differences in activity levels. Altogether, our results suggest that even one extreme event can have long-term consequences for reproductive success and may influence subsequent generations.

In parenting endotherms, high ambient temperatures in conjunction with metabolic demands of parenting can lead to reduced body condition, leading to an inability to meet offspring requirements via provisioning or lactation (Nilsson and Nord 2018; Khera et al. 2023). Ectotherms, on the other hand, may be able to increase behaviors with high metabolic demands under increased ambient temperatures until upper thermal tolerance is reached (Dillon et al. 2010; Sinclair et al. 2016). In stickleback, previous work has found that fanning behaviors increased under sustained increased temperature (Hopkins et al. 2011; Isotalo et al. 2022), while body condition decreased, possibly due to the high energy demands of parenting (Isotalo et al. 2022). Here, we found that stickleback males from this population were not impacted by the heat wave regarding their mass or body condition, suggesting heat itself was not sufficient to induce condition-dependent changes over 5 d or across the parenting cycle. However, in contrast to previous studies investigating parenting under high temperature, males that had experienced the heat wave reduced the percent of time fanning their eggs while at the nest, beginning on day 3 post-fertilization (when metabolic demands of embryos begin to increase (Van Iersel 1953)) and continuing throughout the entirety of the nesting cycle. This suggests that while males exposed to a heat wave were still able to be at their nest, they may have experienced metabolic stress resulting in lower energy reserves, leaving them unable to maximize fanning. Considering males began parenting from 2 to 38 d post-heat wave, this suggests significant long-term implications for brief heat exposure on crucial reproductive behaviors. Alternatively, males exposed to a heat wave may be altering their reproductive strategy by reserving energy during the current reproductive bout to maximize energy for the second, in a tradeoff between somatic maintenance and reproductive investment (Ratz et al. 2024). In conjunction with other stickleback studies demonstrating different responses to heat depending on when it occurs during or preceding a nesting cycle (Isotalo et al. 2022), our results highlight the importance of measuring response to extreme events across multiple time points and add to a growing body of evidence that even if heat waves may not directly affect long-term condition, they can have significant reproductive costs.

Heat stress can elevate glucocorticoid levels across taxa, potentially imposing energetic or immunological costs (Pankhurst 2011; Jessop et al. 2016; de Bruijn and Romero 2018; Mentesana and Hau 2022). Contrary to our predictions that heat stress would elevate glucocorticoids after a heat wave, we instead found that control males had increased cortisol compared to males exposed to a heat wave. Importantly, while cortisol is typically associated with physiological stress, it is also associated with initiating territoriality and aggression in stickleback males, with a peak in cortisol during territory and nest-building phases (Sebire et al. 2007). A rise in cortisol may therefore be associated with preparation for reproduction and parenting. In this context, the rise in cortisol levels in control males could align with control males exhibiting more readiness for territory establishment and nest building as compared to heat wave males. This is further reflected in longer (but non-significant) latency to build a nest in heat-wave males. Males exposed to a heat wave may, therefore, have a dampening of cortisol responses. This suggests that males exposed to brief periods of elevated temperatures may experience inhibition regarding typical behaviors aligned with territoriality and aggression that are important in the context of reproduction and parental care, which may have implications that lessen the motivation to reproduce. Whether heat actively suppresses cortisol in male threespine stickleback, or whether heat reduces behavioral initiation of territoriality subsequently reflected in cortisol response, remains to be seen.

Heat can both directly (via damage to gametes) or indirectly (via changes to secondary sexual characteristics or parental behavior) impact male reproductive success. Our finding that males exposed to a heat wave had embryos with a longer latency to hatch and had reduced hatching success may, therefore, be influenced by heat-wave-induced changes in paternal behavior across the nesting cycle, or via effects on male fertility (Mehlis and Bakker 2014; Breedveld et al. 2023; Ratz et al. 2024). We found no correlation between fanning and reproductive success, suggesting that our findings may be driven by direct effects on male gametes. Chronic heat exposure in adulthood is known to damage sperm and negatively influence sperm performance (Nguyen et al. 2013; Gasparini et al. 2018; Wang and Gunderson 2022). Recently, even short-term exposure to heat in heat waves have been shown to lower quality of sperm and fertilization success across taxa, including in fishes (Hurley et al. 2018; Sales et al. 2018; Leach et al. 2021; Breedveld et al. 2023; Ratz et al. 2024). Importantly, male stickleback suppress spermatogenesis during the breeding season (Borg 1982; Zbinden et al. 2001); therefore, any heat damage to testes or sperm during this time will be reflected in all subsequent fertilizations. This highlights how timing, species-specific morphology, and life histories can increase the susceptibility of populations to extreme environmental events. Future work examining sperm viability, morphology, and quantity post-heat exposure in stickleback will help tease apart these potential mechanisms of lowered reproductive success and whether these changes can be reversed when spermatogenesis resumes in the winter.

Non-genetic paternal effects on offspring growth are seen across taxa (Wong et al. 2007; Eilertsen et al. 2008; Adler and Bonduriansky 2013; Stein and Bell, 2014; Stein et al. 2018). While offspring of heat wave males were not smaller nor weighed less, they were in worse body condition. Ambient heat during development often results in smaller size and lower body condition for offspring (Corregidor-Castro and Jones, 2021; Oswald et al. 2021; Bourne et al. 2022; Eastwood et al. 2023; Khera et al. 2023); in fishes and other ectotherms, this may be due to heat decreasing development time (Allen and Wootton 1982; Atkinson 1994; Arendt 2011; L. Trip et al. 2014). Importantly, all offspring in our study were reared at 18°C and provided the same food resources and space relative to rearing density; therefore, changes to offspring are most likely to be due to paternal influence. As Fulton’s K estimates fat reserves in small fishes (Kotrschal et al. 2011), this suggests that offspring of heat-wave males are less able to maintain fat than offspring of control males. Whether this is due to paternal behavioral effects or damage to sperm on development cannot be disentangled here but provides an intriguing pathway for future investigations into the interplay between behavioral and physiological responses to heat wave conditions on offspring development.

We found no direct effects of paternal exposure to heat on offspring behavior. Rather, offspring’s behavior in the anxiety-related scototaxis assay was primarily impacted by body condition or rearing density. As hatching success and offspring body condition were influenced by paternal exposure to heat waves, this suggests the potential for indirect effects on subsequent generations. Time in the pod prior to emergence into a novel environment was influenced by rearing density, where offspring of larger clutches emerged faster into a novel arena, which may be considered a risk-taking behavior. There was natural variation in density related to both variations in initial egg mass and in hatching success. As fathers exposed to a heat wave had lower hatching success, this has the potential to impact future risk-taking behaviors via early life social interactions with siblings. In this study, we reared offspring in sibling groups to maintain family identity. While this confounds genetic and tank effects, we found no effect of paternal ID/tank. However, future work teasing apart genetic vs rearing effects will be important for identifying underlying causes of generational effects of heat. Similarly, juveniles with worsened body condition swam more in the behavioral assay, suggesting that a previous exposure to a heat wave could impact energetic loads of offspring—indeed, this result presents the possibility that lower fat reserves (lower Fulton’s *K*) in offspring of heat-wave fathers may be due to increases in activity rather than physiological processes affecting fat storage. Altogether, our results on offspring behavior demonstrate the potential for indirect effects of paternal experience to have long-term impacts on offspring under historically standard thermal conditions.

In this study, we were interested in testing whether one relatively short-term event would have repercussions for both parents and offspring even after conditions returned to “normal”; therefore, we did not raise nor measure parenting or offspring behavior under stressful temperature regimes. If parental effects are adaptive, offspring may not show differences under mismatched conditions (e.g. heat-wave father, control personal experience) but would show differences compared to control under conditions that match parental experience. Indeed, parental effects are often strongly context-dependent, such that changes to offspring traits, performance, or fitness may not be observable depending on the environment in which they are measured (Rossiter 1996; Marshall and Uller 2007; Uller et al. 2013). For example, in sheepshead minnows (*Cyprinodon variegatus*), offspring raised under the same thermal conditions as their parents showed higher growth rates than offspring raised under mismatched conditions (Salinas and Munch 2012; Munch et al. 2021). It is possible that parental exposure to heat may influence offspring behavior in the scototaxis assay if conducted at higher temperatures. Understanding whether long-term consequences of parent exposure to heat on offspring are adaptive or maladaptive will require measuring offspring behavior and reproductive performance across multiple thermal contexts.

Our study population has been experiencing heat waves with increasing frequency and intensity over the two decades preceding our data collection (Gershunov and Guirguis 2012; Philip et al. 2022; Thompson et al. 2022). Explanations for our findings that post-heatwave males showed changes in embryo development time (latency to hatch) and reduced fanning across the nesting cycle may, therefore, not reflect gametic damage or energetic exhaustion, but instead, an adaptive response. For example, reduction in fanning may indicate tradeoffs between current and future reproductive effort: if males that reduce fanning are better able to survive a subsequent heat wave, they may be better able to complete a second nesting cycle after that heatwave ends compared to males that do not adjust their fanning behavior. In a study examining paternal effort and reproductive success across two heat bouts, males that parented at high levels during the first heat wave showed significantly reduced condition, parenting ability, and reproductive success during the second (Isotalo et al. 2022). A longer development time may allow offspring to compensate for reductions in fanning by lowering energetic requirements. Comparisons of parenting, reproduction, and embryo development across populations with a history of extreme events to those that have remained relatively unaffected (e.g. spring-fed rivers better able to maintain lower water temperatures) would be valuable for both understanding selective pressures underlying parental care evolution and for informing predictions of population persistence.

Overall, our results show strong support for heat waves influencing paternal care, reproductive success, and offspring development in a population experiencing extreme heat events, even after conditions return to normal. These data highlight the importance of studying how ecologically relevant stressors influence plasticity in parental behavior and show that these effects can cascade across generations. This complements recent work in stickleback demonstrating that while an increase in ambient temperature may initially increase parental behaviors and reproductive success, long-term consequences of heat eliminate these effects (Isotalo et al. 2022). In addition, investigating the effects of heatwaves on reproduction can provide valuable information for population persistence, as thermal thresholds for fertility are often lower than for survival (Walsh et al. 2019; Parratt et al. 2021) and may cascade to lower reproductive success and survival for offspring (Gasparini et al. 2018; Sales et al. 2018; Ratz et al. 2024). A goal of future studies will be to understand the mechanisms underlying these long-term effects, the interplay between offspring requirements and parental ability to adjust behavior to meet these needs, and how we may use these together to predict population response to extreme environmental changes.

## Supplementary Material

arae036_suppl_Supplementary_Figures_S1-S2

## Data Availability

Analyses reported in this article can be reproduced using the data provided by Barrett and Stein (2024).
