# Supplementary material for: Short-term heat waves have long-term consequences for parents and offspring in stickleback
Source: Behav Ecol. 2024 Apr 27;35(4):arae036. doi: 10.1093/beheco/arae036 (PMC11110458; doi:10.1093/beheco/arae036)
Supplement: arae036_suppl_Supplementary_Figures_S1-S2 [file arae036_suppl_supplementary_figures_s1-s2.docx]

SUPPLEMENTAL FIGURES


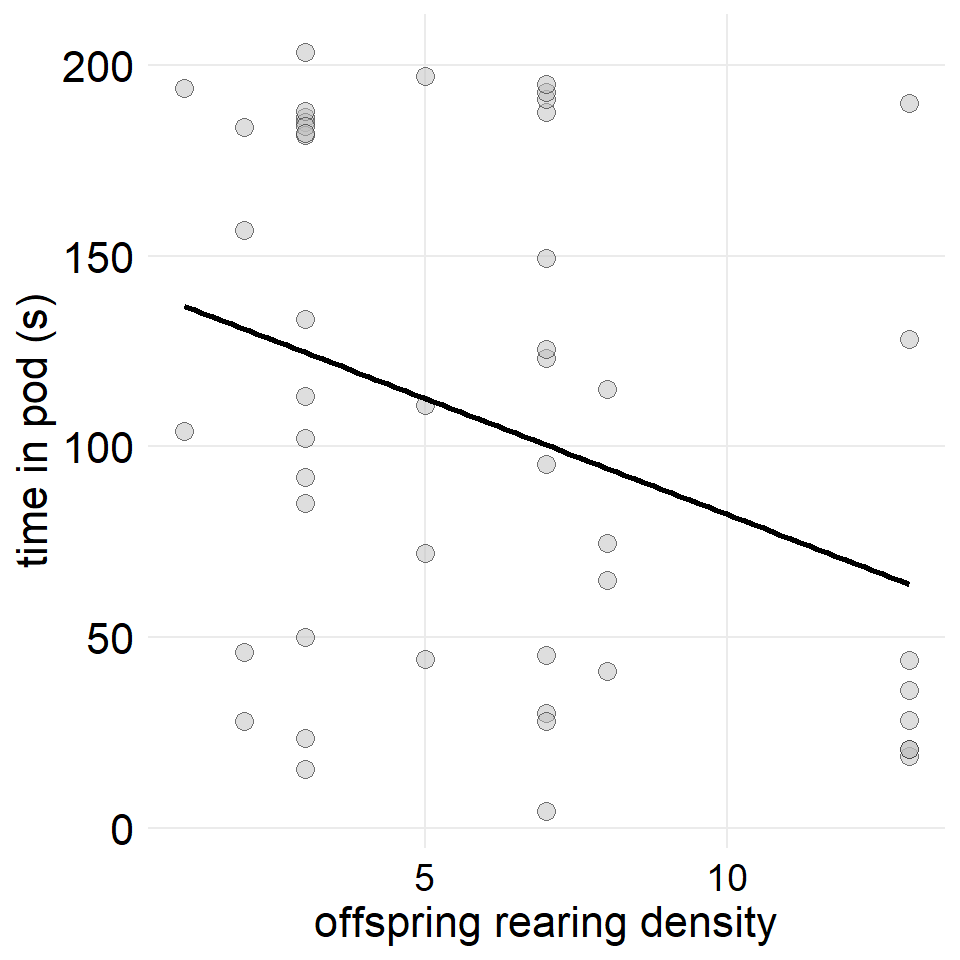


**Fig S1.** Offspring rearing density influenced time in pod in a scototaxis assay. Offspring reared at higher densities emerged from the pod faster than offspring reared at lower densities.


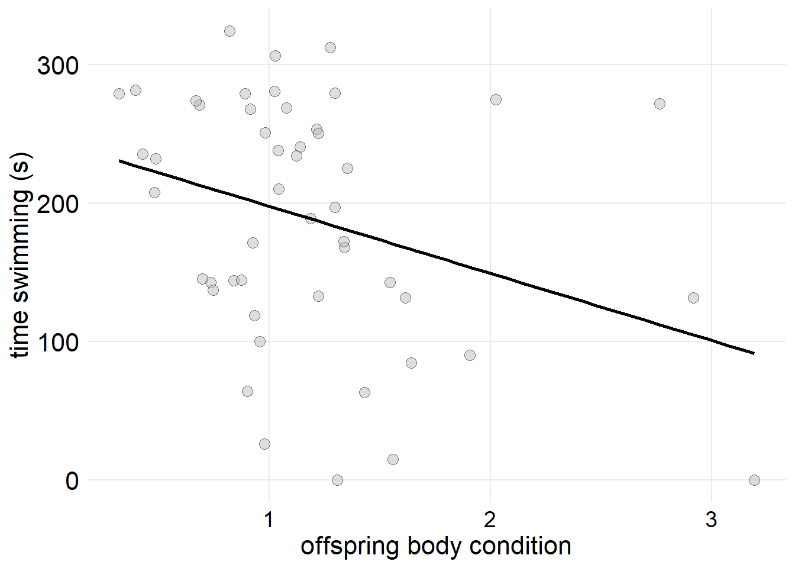


**Fig S2.** Offspring body condition (Fulton’s *K*, g/mm^3^ * 100,000) influenced time swimming in a scototaxis assay. Offspring in better body condition swam less than offspring in poor body condition.
